# Low myoglobin concentration in skeletal muscle of elite cyclists is associated with low mRNA expression levels

**DOI:** 10.1007/s00421-023-05161-z

**Published:** 2023-03-06

**Authors:** Nina Jacobs, Daniek Mos, Frank W. Bloemers, Willem J. van der Laarse, Richard T. Jaspers, Stephan van der Zwaard

**Affiliations:** 1 Department of Human Movement Sciences, Amsterdam Movement Sciences, Vrije Universiteit Amsterdam, Van der Boechorststraat 7, 1081 BT Amsterdam, The Netherlands; 2 Laboratory for Myology, Department of Human Movement Sciences, Amsterdam Movement Sciences, Vrije Universiteit Amsterdam, Amsterdam, The Netherlands; 3 Department for Trauma Surgery, Amsterdam UMC, Amsterdam, The Netherlands; 4 Department of Physiology, Amsterdam UMC, Amsterdam, The Netherlands

**Keywords:** Oxygen transport, Muscle biopsy, Muscle fiber type, Cycling, Myonuclei, Satellite cells

## Abstract

Myoglobin is essential for oxygen transport to the muscle fibers. However, measurements of myoglobin (Mb) protein concentrations within individual human muscle fibers are scarce. Recent observations have revealed surprisingly low Mb concentrations in elite cyclists, however it remains unclear whether this relates to Mb translation, transcription and/or myonuclear content. The aim was to compare Mb concentration, Mb messenger RNA (mRNA) expression levels and myonuclear content within muscle fibers of these elite cyclists with those of physically-active controls. Muscle biopsies were obtained from *m. vastus lateralis* in 29 cyclists and 20 physically-active subjects. Mb concentration was determined by peroxidase staining for both type I and type II fibers, Mb mRNA expression level was determined by quantitative PCR and myonuclear domain size (MDS) was obtained by immunofluorescence staining. Average Mb concentrations (mean ± SD: 0.38 ± 0.04 mM vs. 0.48 ± 0.19 mM; *P* = 0.014) and Mb mRNA expression levels (0.067 ± 0.019 vs. 0.088 ± 0.027; *P* = 0.002) were lower in cyclists compared to controls. In contrast, MDS and total RNA per mg muscle were not different between groups. Interestingly, in cyclists compared to controls, Mb concentration was only lower for type I fibers (*P* < 0.001), but not for type II fibers (*P* > 0.05). In conclusion, the lower Mb concentration in muscle fibers of elite cyclists is partly explained by lower Mb mRNA expression levels per myonucleus and not by a lower myonuclear content. It remains to be determined whether cyclists may benefit from strategies that upregulate Mb mRNA expression levels, particularly in type I fibers, to enhance their oxygen supply.

## Introduction

Oxygen (O_2_) transport to the mitochondria is critical to achieve an exceptional endurance performance (van der Zwaard et al. 2021). Within the contracting muscle fibers, myoglobin (Mb) may facilitate over ~ 50% of the oxygen transport from the sarcolemma to the mitochondria at heavy to severe exercise (Wittenberg and Wittenberg 2003; Gros et al. 2010; Poole et al. 2022). This O_2_ is subsequently used to resynthesize the adenosine triphosphate (ATP) necessary to sustain the endurance performance for a prolonged duration. It is presumed that Mb concentration is upregulated in mammals that undergo extended periods of apnea when diving (Ordway and Garry 2004) and in response to the O_2_ demand within the mitochondria (Wittenberg and Wittenberg 2003). Although low or absent Mb concentrations do not necessarily lead to impaired exercise capacity (Garry et al. 1998; Gödecke et al. 1999), improved Mb concentrations may still exert a critical role in the supply of O_2_ to the mitochondria when optimizing endurance performance, attributing to the aerobic capacity of individual muscle fibers.

In humans, there are only few studies that have assessed concentrations of Mb protein within the individual muscle fibers (van der Zwaard et al. 2021). Quantitative histochemistry of biopsy sections with a vapor-fixation technique (Lee-de Groot et al. 1998; van Beek-Harmsen et al. 2004) allows to determine Mb protein concentration in individual skeletal muscle fibers. An advantage of this approach is that it allows for detection of fiber-type specific differences in Mb concentration that are masked in homogenized muscle samples (Bekedam et al. 2009). Young elite cyclists showed an average Mb concentration of 0.38 ± 0.04 mM (mean ± SD; (van der Zwaard et al. 2018b)) in their individual muscle fibers, which is surprisingly lower than concentrations of ~ 0.50–0.60 mM measured in young elite hockey players and older healthy controls and cardiac patients (Bekedam et al. 2009; van der Zwaard et al. 2018a), despite the ability to deliver an exceptional physical performance (van der Zwaard et al. 2018b). One explanation for the lower Mb concentrations may be that Mb is downregulated in elite cyclists to accommodate for a strong iron demand during hypoxia-induced erythropoiesis (Robach et al. 2007). However, red blood cell and hematocrit values did not indicate this in the young elite cyclists (van der Zwaard et al. 2018b). Another explanation could be the regulation of transcription and translation of Mb. So far, however, little is known about the regulation of Mb concentration in human muscle fibers and why this Mb concentration may be lower in elite cyclists.

Insights into the transcription and translation of Mb is required to determine limiting factors for the regulation of Mb concentration. The total mRNA of Mb that is transcribed in the muscle fiber does not only depend on the mRNA expression within each myonucleus, but also on the number of myonuclei and their corresponding myonuclear domain size (MDS), i.e. the volume of cytoplasm for which one myonucleus supplies the necessary gene transcripts. A lower MDS indicates a larger potential for transcription per volume muscle tissue (van Wessel et al. 2010), since each nucleus supplies a smaller volume of cytoplasm. MDS can be lowered by myonuclear addition, such as mediated by satellite cells (Petrella et al. 2008). Last, during the translation of Mb, the available mRNA is converted into Mb protein in which subsequently a heme molecule is incorporated. Currently, it remains to be determined whether the low Mb concentration values in elite cyclists are because of limitations in Mb translation, transcription and/or myonuclear content.

The aim of this study was to compare the Mb concentration, Mb mRNA expression levels and myonuclear content between elite cyclists and physically-active controls. Considering previous observations in this cohort of cyclists (van der Zwaard et al. 2018b), we hypothesized that Mb concentration is lower in the cyclists compared to physically-active controls because of a lower Mb mRNA expression and/or a larger myonuclear domain size.

## Methods

### Participants

A total of 29 cyclists and 20 physically-active controls participated in this study. Characteristics of the controls (means ± SD) were: age 34 ± 8 years, height 1.84 ± 0.08 cm, weight 93.3 ± 10.7 kg, BMI 27 ± 2. Characteristics of the cyclists were: age 26 ± 7 years, height 1.86 ± 0.06 m, weight 77.3 ± 8.0 kg, BMI 22 ± 2 and included track sprint, team pursuit, and road cyclists. Training characteristics of the cyclists can be found https://faseb.onlinelibrary.wiley.com/action/downloadSupplement?doi=10.1096%2Ffj.201700827R&file=fsb2fj201700827r-sup-0001.docx. Cyclists competed at the national, international, or Olympic level, except for 4 amateur road cyclists. Note that data from 28 cyclists within this cohort of participants has previously been reported (van der Zwaard et al. 2016, 2018b), leading us to the research question of the present study. Of note, only the average myoglobin protein concentrations and muscle fiber type distribution from these elite cyclists have been published before. All participants were 18 years or older and provided written informed consent after experimental procedures and risks of the study were explained to them. The study was conducted according to the Declaration of Helsinki (2013) and was approved by the medical ethics committee of the VU Medical Center, Amsterdam, the Netherlands (NL43423.029.13 and NL49060.029.14).

### Muscle biopsy sampling

From both groups, skeletal muscle biopsies of the m. vastus lateralis muscles were obtained (~ 15 cm above the patella at a depth of ~ 4 cm) under local anesthesia with the modified Bergström technique with suction (Tarnopolsky et al. 2011). Participants were not allowed to perform any physical exercise the day before the biopsies were obtained. Biopsies were stored in liquid nitrogen after alignment with fiber arrangement and positioning fibers at slack length. Subsequently, biopsy samples were cut in transversal sections (10 µm thick) making use of a cryostat (−20 °C). The sections were collected on polylysine-coated slides and stored at −80 °C until further use.

### Quantitative histochemistry

To determine Mb concentration within individual muscle fibers, frozen muscle biopsy sections were fixated using a vapor-fixation technique and a 2.5% glutaraldehyde buffer (to prevent loss of Mb (van Beek-Harmsen et al. 2004)), and incubated as described in detail previously (Bekedam et al. 2009; van der Zwaard et al. 2018a, b). After incubation, Mb concentration was determined while excluding peroxidase activity of hemoglobin and after calibration with gelatin blocks containing a known concentration of equine Mb (Lee-de Groot et al. 1998). Images were captured with $\times$10 objective using a CCD camera (Sony XC77CE, Towada, Japan) connected to a LG-3 frame grabber (Scion, Frederick, MD) and a DMRB microscope with calibrated gray filters (Leica, Wetzlar, Germany). As Mb concentrations are known to differ between fiber types, Mb concentration was determined for type I and type II fibers separately. Mb concentration was obtained from 436-nm absorbance measurements in 20 randomly selected cells for each fiber type using the ImageJ software package (National Institutes of Health, Bethesda, Maryland, USA). Subsequently, the Mb concentrations in type I and type II fibers were used to calculate the weighted average Mb concentration based on the relative muscle fiber type distribution of each participant (i.e. average Mb concentration = [Mb] in type I fibers $\times$ percentage type I fibers + [Mb] in type II fibers $\times$ percentage type II fibers).

Fiber type was determined by immunofluorescence staining for myosin heavy chain isoforms (Bloemberg and Quadrilatero 2012). Sections were blocked with 10% normal goat serum and incubated with monoclonal primary antibodies against type I (1 $\upmu$g/ml BA-D5, Developmental Studies Hybridoma Bank, Iowa City, IA), and type II fibers (1 $\upmu$g/ml SC-71, Developmental Studies Hybridoma Bank, Iowa City, IA). Subsequently, incubation in the dark was performed using secondary fluorescent antibodies for type I (5 $\upmu$g/ml Alexa Fluor 488 IgG_2b_) and type II fibers (5 $\upmu$g/ml Alexa Fluor 488 IgG_1_). Images were captured using a $\times$10 objective and a CCD camera (PCO; Sensicam, Kelheim, Germany) connected to a fluorescence microscope (Axiovert 200 M; Zeiss, Göttingen, Germany), with the image processing software (Slidebook 5.5; Intelligent Image Innovations, Denver, CO). Fiber type was determined in at least 200 cells (if present).

Furthermore, transversal and longitudinal sections were stained for myonuclei, satellite cells and basal lamina. Myonuclei were stained using 4',6-diamidino-2-phenylindole (DAPI, Molecular Probes) in Vectashield. Staining of satellite cells was performed using the primary antibody mouse monoclonal Paired Box 7 (PAX7) (2.8 µg/ml) in Phosphate Buffered Saline (PBS)/0,1% Bovine Serum Albumin (BSA) and the secondary antibody Alexa 488 IgG1 goat anti-mouse (1:100 in PBS/0,1%BSA), in the dark. Staining of the basal lamina was performed by incubating the samples with Wheat Germ Agglutinin (WGA) (1:50 in PBS). Images of the sections were captured at $\times$20 objective using a CCD camera connected to the fluorescence microscope. Image size was calibrated taking into account the pixel aspect ratio. Fiber cross-sectional area (FCSA) was determined from manual segmentation tracing the basal lamina in randomly selected fibers in ImageJ, excluding fibers with a circularity of 0.60 or lower (Kim et al. 2007). Circularity of muscle fibers was calculated as follows (Verdijk et al. 2009):

1 $${\text{Circularity }} = \frac{{4\pi \times {\text{FCSA}}}}{{{\text{perimeter}}^{2} }}$$

where FCSA is the fiber cross-sectional area in µm^2^ and the perimeter is in µm. Also, the numbers of myonuclei and satellite cells were determined in circular cells only, to avoid overestimation of the myonuclear domain. Myonuclear fragments were identified from stained biopsy sections as DAPI + spots located against the basal lamina, unless these spots also stained positive for Pax7. In this case, these spots were considered to be satellite cells. DAPI + spots located in between basal laminas were considered fibroblasts, endothelial cells or macrophages. Finally, DAPI + spots located within the cytoplasm (not in contact with the basal lamina) were considered to be central nuclei. Satellite cells, fibroblasts, endothelial cells or macrophages and central nuclei were not counted as myonuclei. FSCA, the number of myonuclear fragments per muscle fiber cross-section and the number of satellite cells per muscle fiber cross-section were determined in 200 circular cells (if present; on average 436 ± 275). From these, we calculated the number of satellite cells relative to the total number of nuclei (number of satellite cells per fiber / [number of myonuclei per fiber + number of satellite cells per fiber] $\times$ 100%) as well as the myonuclear domain size (MDS) in accordance with the formulas below (van der Meer et al. 2011)

2 $${\text{MDS}} = \frac{{{\text{FCSA}}}}{{{\text{N}}_{{{\text{m}},{\text{l}}}} }}$$

3 $${\text{N}}_{{\text{m,l}}} \, = \,\frac{{{\text{N}}_{{\text{m,f}}} \times \,{\text{L}}_{{\text{f}}}}}{{({\text{D}}_{{\text{s}}} \times \,{\text{F}})}}$$

Where MDS is myonuclear domain size (in pL), FCSA is the fiber cross-sectional area (in µm^2^), and N_m,l_ is the number of myonuclei per mm of given muscle fiber length. Here, N_m,l_ is calculated according to formula 3, where N_m,f_ is the number of myonuclear fragments per muscle fiber cross-section, L_f_ is the length of the muscle fiber segment (i.e. 1000 µm) and D_s_ is the thickness of the biopsy cryosection (i.e. 10 µm). F is the correction factor for multiple counting of the myonuclei. From longitudinal biopsy sections, the average myonuclear length of participants was determined (11 ± 2 µm). Given the smallest myonuclear fragment that can be detected by DAPI is 1 µm (van der Meer et al. 2011), every nucleus can be identified in two consecutive biopsy sections of 10 µm. This means that, on average, myonuclei are always counted twice as these are present in two consecutive biopsy sections. Therefore, correction factor F was set to 2.

### Quantitative polymerase chain reaction

To obtain Mb messenger RNA (mRNA) and total RNA expression levels, muscle biopsy samples were homogenized and real-time quantitative Polymerase Chain Reaction (qPCR) was performed. First, muscle biopsies were cut in smaller tissue samples in the cryostat (−20 °C) and were weighed while frozen. The samples were homogenized in TRI reagent and kept at room temperature for 5 min. RNA was isolated (using RNAqueous^®^-Micro Kit in cyclists and RiboPure Kit in controls). Total RNA per µl elution buffer was determined to calculate total RNA per mg muscle. Reverse Transcription PCR was used to transcribe the obtained RNA strands into its DNA complements. The resulting cDNA was amplified using real-time qPCR. Relative Mb mRNA expression levels were quantified with qPCR based on its cycle threshold (CT), using the following SYBR green primers: 5’–AATGGCAGTTG-GTGCTGAAC-3’ (forward primer) and 5’–GGTGACCCTTAAAGAGCCTGAT-3’ (reverse primer). Mb mRNA expression levels were determined relative to expression levels of the housekeeping gene ribosomal 18S RNA to correct for total RNA concentrations. 18S expression levels were quantified with qPCR, using Taqman Universal Mastermix (4,304,437 life Technologies). Subsequently, Mb mRNA expression levels were calculated with respect to 18S expression levels, using their corresponding cycle thresholds (i.e. ${2}^{-\Delta \mathrm{CT}}$, where $\Delta$CT is CT_Mb_–CT_18S_).

### Statistical analysis

Values are presented as means ± standard deviations (SD). Normality was evaluated using Shapiro–Wilk tests and homogeneity by the Levene’s test. Group differences between cyclists and physically-active controls were evaluated with the independent samples *T*-test or non-parametric Mann–Whitney *U* test for weighted average Mb concentration, Mb mRNA expression levels, total RNA concentration per mg muscle, MDS, FCSA, muscle fiber type distribution, the number of myonuclei per fiber, the number of satellite cells per fiber and the number of satellite cells relative to the total number of nuclei. Subsequently, differences in Mb concentration in type I and type II fibers were tested using the independent samples *T*-test or non-parametric Mann–Whitney *U* test and *P*-values were adjusted using Bonferroni corrections for multiple testing. The correlation between Mb concentration and Mb mRNA expression levels was determined with a Spearman correlation, after pooling participants into one group. Findings were considered to be significant when *P* < 0.05.

## Results

### General

**Fig. 1:**
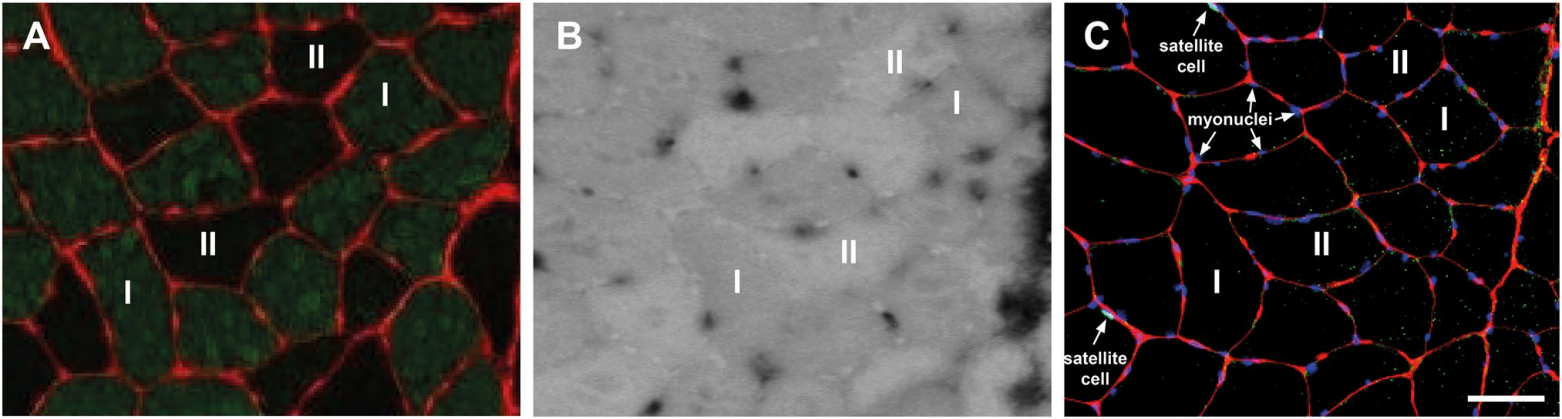
Immunohistochemical staining for myofibrillar myosin heavy chain type I expression **A**, peroxidase staining for myoglobin concentration **B** and immunohistochemical staining for myonuclear and satellite cell fragments **C** from an elite cyclist. Histochemical assays for myoglobin contain black spots because of peroxidase activity of hemoglobin in the capillaries, which were excluded from analysis. Scale bar is 100 µm

Figure 1 shows a typical example of individual muscle fibers that were stained for myoglobin, fiber type, myonuclei and satellite cells. Histochemical assays for myoglobin contain black spots because of peroxidase activity of hemoglobin in the capillaries, which were excluded from the assessment of myoglobin concentration.

### Myoglobin expression

**Fig. 2:**
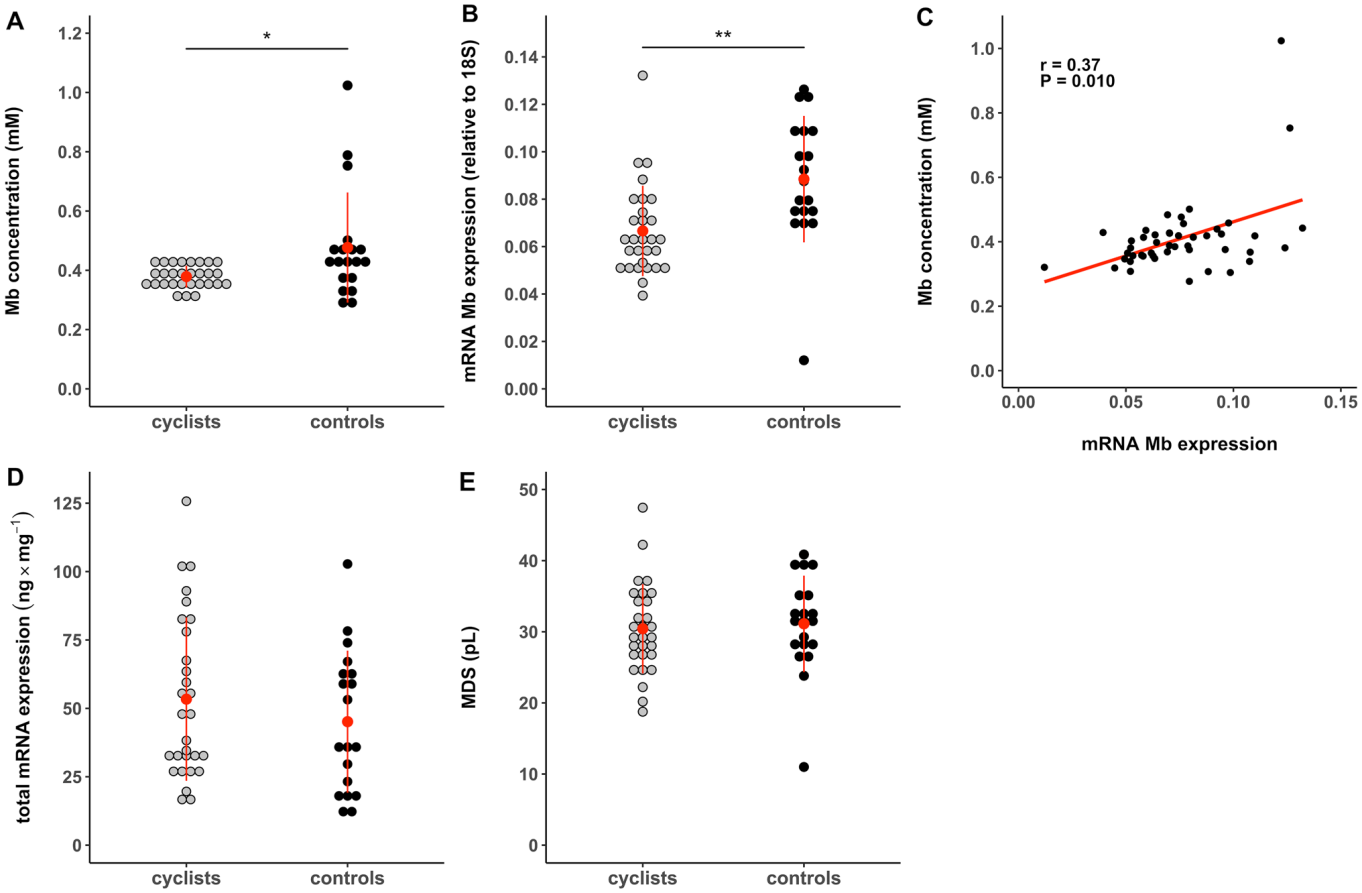
Average Mb concentration and Mb mRNA expression levels were lower in cyclists compared to physically-active controls, whereas total RNA concentrations and MDS were not different. Values are means ± SD. **A** Mb concentration (mM). **B** Mb mRNA expression level (relative to 18S). **C** Relationship between Mb mRNA expression level and Mb concentration in the pooled group of cyclists and controls (r_s_ = 0.37, *P* = 0.010). **D** Total RNA concentration per mg muscle (ng/mg) and **E** MDS in pL. Mb: myoglobin; MDS: myonuclear domain size. * is *P* < 0.05, ** is *P* < 0.01

Figure 2A shows that the cyclists had a 21% lower average Mb concentration within their muscle fibers compared to controls (0.38 ± 0.04 mM vs. 0.48 ± 0.19 mM, *P* = 0.014). To explain the lower Mb concentration in the cyclist group, Mb mRNA expression levels within the muscle fibers were determined relative to housekeeping gene 18S. These Mb mRNA expression levels relative to that of 18S RNA were also 24% lower in cyclists compared to controls (0.067 ± 0.019 vs. 0.088 ± 0.027, *P* = 0.002, Fig. 2B). In accordance with these observations, pooling data from both cyclists and controls showed a significant correlation between Mb concentration and Mb mRNA expression levels relative to 18S (r = 0.37, *P* = 0.010). The total RNA content per mg muscle tissue did not differ between cyclists and control subjects (53.4 ± 29.9 ng/mg vs. 45.1 ± 26.0 ng/mg, *P* = 0.399, Fig. 2C). Altogether, the results suggest that the low Mb concentration in muscle fibers of elite cyclists is accompanied by low Mb mRNA expression levels (explaining 14% of the variance).

**Fig. 3:**
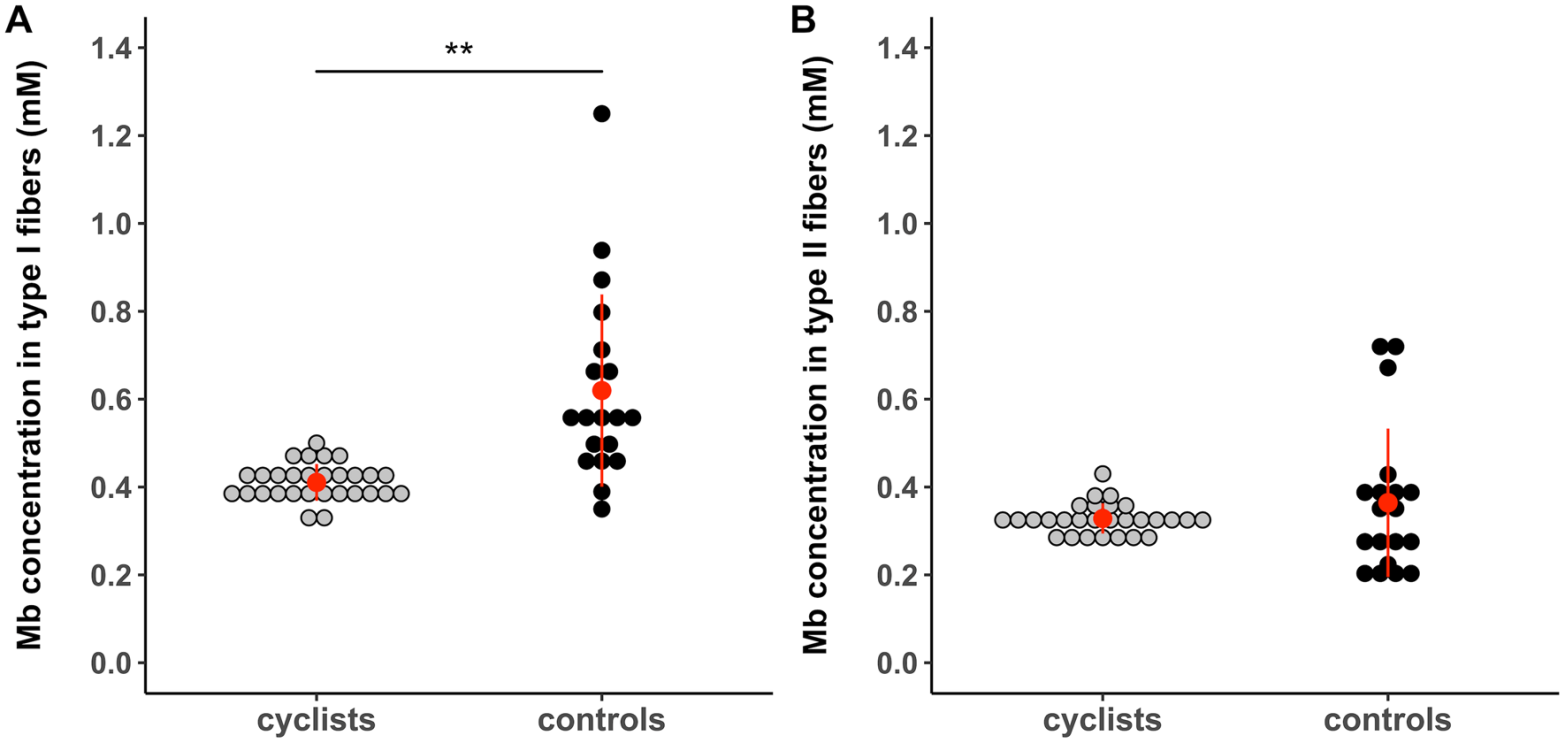
Compared to physically-active controls, elite cyclists showed lower Mb concentration in type I, but not in type II muscle fibers. Values are means ± SD. **A**: Mb concentration in type I muscle fibers (mM). **B**: Mb concentration in type II muscle fibers (mM). Mb: myoglobin; * is *P* < 0.05, ** is *P* < 0.01

Interestingly, Mb concentration was only lower for type I fibers (*P* < 0.001), but not for type II fibers (*P* > 0.05) in cyclists compared to controls (Fig. 3). The average myoglobin content was ~ 25% higher in type I than in type II fibers for cyclists, whereas myoglobin content was ~ 84% higher in type I compared to type II fibers for controls (25 ± 9% vs. 84 ± 54%, *P* < 0.001).

### Myonuclear content

The lower Mb mRNA expression levels could relate to lower myonuclear content in the elite cyclists, i.e. a lower myonuclear density or a larger MDS. Nonetheless, our data show that both groups had similar myonuclear fragments per muscle fiber cross-section (see Table 1) and also the MDS did not differ between cyclists and control subjects (30.4 ± 6.3 pL vs. 32.7 ± 8.8 pL, *P* = 0.287, Fig. 2D). Even the number of satellite cells per muscle fiber, the precursor of new myonuclei, was similar between both groups (Table 1). These results indicate that the lower Mb mRNA expression level in elite cyclists was likely not due to a lower myonuclear content (i.e. lower potential for transcription).

**Table 1:** Myonuclear content, muscle fiber type distribution and fiber cross-sectional areas of elite cyclists and physically-active controls obtained from biopsy cross-sections

	Cyclists	Controls	*P-*value
FCSA type I fibers (µm^2^)	6,469 ± 1,600	6,742 ± 1,433	0.543
FCSA type II fibers (µm^2^)	6,724 ± 1,813	6,891 ± 1,531	0.739
MHC type I fibers (%)	66.0 ± 13.7*	45.2 ± 10.6	< 0.001
MHC type II fibers (%)	34.0 ± 13.7*	54.8 ± 10.6	< 0.001
Myonuclei per fiber	4.34 ± 0.76	4.30 ± 0.83	0.856
Satellite cells per fiber	0.13 ± 0.05	0.15 ± 0.05	0.166
Satellite cells per myonuclei (%)	2.99 ± 1.28	3.49 ± 0.85	0.021

*FCSA* fiber cross-sectional area, *MHC* myosin heavy chain

## Discussion

The aim of this study was to compare Mb protein concentration within muscle fibers, Mb mRNA expression and myonuclear content between elite cyclists and physically-active control subjects. The present findings show that the average Mb concentration was lower in the elite cyclists, which is associated with their lower Mb mRNA expression levels and not explained by differences in their myonuclear content (i.e. myonuclei, satellite cells and myonuclear domain size). Interestingly, Mb concentration levels were only lower for type I muscle fibers, but not for type II muscle fibers. In summary, our results show that the lower Mb concentration in elite cyclists, particularly within type I muscle fibers, may be partially explained by the lower Mb mRNA expression level and not by the potential for transcription of the myonuclei.

Mb protein concentration of individual muscle fibers was lower in the elite cyclists compared to physically-active controls (0.5 mM) and to that in populations as reported in previous literature (0.5–0.6 mM; (Bekedam et al. 2009; van der Zwaard et al. 2018a)), even though cyclists had a higher percentage of type I fibers compared to controls. The lower average Mb concentration is mainly explained by the lower Mb concentration in type I fibers, and not by that in type II fibers, in the cyclists. Whereas Mb concentration is typically ~ 50% higher in human type I compared to type II muscle fibers (Möller and Sylvén 1981; Jansson and Sylvén 1983; Nemeth and Lowry 1984; van der Zwaard et al. 2018a), this was only 25% higher in our elite cyclists. These lower Mb concentrations have functional implications for Mb’s ability to store and transport O_2_ within the muscle fibers (Wittenberg and Wittenberg 2003; Gros et al. 2010; Poole et al. 2022). First, Mb desaturates immediately after the onset of exercise (Chung et al. 2005), releasing its stored O_2_ so that the blood flow can adjust to meet the increased metabolic demand. Second, partly desaturated Mb facilitates O_2_ consumption and contractile function during steady-state conditions, as blocking Mb-mediated O_2_ transport using hydrogen peroxide acutely reduced O_2_ consumption and tension generated by the muscle (Cole 1982). Last, at heavy-to-severe exercise intensities—when intracellular PO_2_ values fall below ~ 5 mmHg—Mb-mediated O_2_ transport becomes particularly dominant and exceeds that of free O_2_ diffusion (Richardson et al. 1995, 2001; Gros et al. 2010). Therefore, negative consequences of the lower Mb concentrations in the cyclists are likely most profound at the onset of exercise or during high-intensity exercise.

Along with the lower Mb concentration, Mb mRNA expression was also lower in cyclists compared to controls. Regulation of Mb mRNA expression is coordinated using the calcineurin/Nuclear Factor of Activated T-cell (NFAT) pathway and requires a combination of hypoxic stress and contractile activity to be upregulated (Kanatous and Mammen 2010). In brief, sustained contractile activity induces a calcium (Ca^2+^) influx by the Ca^2+^ release from the sarcoplasmic reticulum, which subsequently activates the phosphatase calcineurin (Stiber and Rosenberg 2011). Calcineurin then dephosphorylates transcription factors Myocyte Enhancer Factor-2 (Mef2) and NFAT (Wu et al. 2000, 2001; Crabtree and Olson 2002; Hogan et al. 2003; Kanatous and Mammen 2010). Upon dephosphorylation, Mef2 and NFAT translocate to the myonucleus and bind to specific sequences on the Mb promoter (A/T motif and NFAT Response Element (NRE), respectively), which stimulates the transcription of Mb mRNA (Garry et al. 2003; Hogan et al. 2003; Kanatous and Mammen 2010). Moreover, peroxisome-proliferator-activated receptor-γ coactivator 1 (PGC-1α)—which regulates expression of type I myosin heavy chain—may also modulate myoglobin transcription via co-activating Mef2 in the presence of calcineurin (Lin et al. 2002). In contrast, Mb mRNA expression is inhibited by the insulin-like growth factor 1 (IGF-1)/AKT/mammalian target of rapamycin (mTOR) signaling pathway (Peters et al. 2017). Specifically, IGF-1—which is a strong anabolic growth factor (Jaspers et al. 2008)—has shown to inhibit Mb mRNA expression by hyperphosphorylation of NFAT by mTOR in C2C12 mouse myotubes (Peters et al. 2017). The lower Mb mRNA expression in our cyclists may therefore relate to factors involved in the calcineurin/NFAT pathway and/or the IGF-1/AKT/mTOR pathway.

Human training studies confirm that Mb mRNA expression levels are upregulated by a combination of contractile activity and hypoxia (Vogt et al. 2001; Brocherie et al. 2018), which is also supported by animal studies in mice (Kanatous et al. 2009) and zebrafish (Jaspers et al. 2014). In the study of Vogt et al. (2001), Mb mRNA expression was only elevated when high-intensity training was performed for 6 weeks in hypoxia but not with high-intensity training in normoxia or training at a lower intensity. Moreover, in elite hockey players that went on a live-high-train-low (LHTL) altitude training camp, Mb mRNA expression levels were only elevated when participants also performed repeated sprint sessions in hypoxia (RSH), but not in normoxia (RSN) (Brocherie et al. 2018). Importantly, also PCG-1α expression increased only in this group that performed LHTL + RSH (Brocherie et al. 2018). Because PGC-1α has been implicated in fiber-type switching towards type I fibers (Lin et al. 2002; Handschin et al. 2007; Zhang et al. 2017) and with elevated Mb mRNA expression levels (Zhang et al. 2017), a reduced PGC-1α expression could potentially explain why Mb concentration was lower in type I but not in type II fibers of our cyclists. Based on the current results and the signaling pathways involved in Mb synthesis, it seems conceivable that elite cyclists did not train (frequently enough) at a sufficiently high intensity with concomitant hypoxia to elicit the desired increases in Mb mRNA or that these increases were transient. Future studies are warranted to investigate the transcription of Mb in more detail, incorporating factors related to the calcineurin/NFAT pathway, the IGF-1/AKT/mTOR pathway.

Myonuclear content did not explain the lower Mb concentration in our cyclists. Skeletal muscle fibers are multinucleated cells, and their potential for transcription is determined by the myonuclear density. Addition of myonuclei occurs with muscle hypertrophy, which happens already from < 10% increases in FCSA onwards (Conceição et al. 2018) and is mediated by satellite-cell activation (Petrella et al. 2008). In contrast, a loss of myonuclei could be observed after denervation-induced atrophy (van der Meer et al. 2011). In the present study, FCSA was similar between cyclists and controls and also the number of myonuclei per fiber, satellite cells per fiber and MDS were not different between both groups. For both groups, myonuclei per fiber and MDS had typical values given their FCSA (Karlsen et al. 2015; Snijders et al. 2020; Hansson et al. 2020), which supports the notion that myonuclei and cellular volume per myonucleus tightly scale with FCSA for the regulation of fiber size. In summary, our results suggest that the lower Mb concentration in the knee extensor muscle fibers of elite cyclist is partly explained by the lower Mb mRNA expression levels (14% explained variance) and not by myonuclear content.

Although it can be expected that higher Mb concentrations would improve the oxygen supply (and utilization) in these elite cyclists, very high Mb concentrations could potentially have harmful detriments, e.g. related to viscosity or oxidative modifications (Mannino et al. 2020). However, for Mb concentration values up to 0.8 mM such detriments have not been reported in humans (Bekedam et al. 2009; van der Zwaard et al. 2018a), which is ~ twofold higher than Mb concentrations in our elite cyclists. Increasing Mb concentrations may also be unneeded. In particular, lower Mb concentrations have been observed in muscles with less diffusion limitation at maximal exercise (Conley et al. 2000) and mice without Mb have a better capillarization (Gödecke et al. 1999). Therefore, lower Mb concentrations could be compensated for by higher capillary-mediated oxygen diffusion during exercise. The capillarization of our cohort of elite cyclists was well-developed (i.e. capillary-to-fiber ratio of ~ 3 and ~ 7 capillaries around the muscle fiber (van der Zwaard et al. 2018b)), similar to that of endurance athletes (van der Zwaard et al. 2021), but higher than that of elite hockey players (i.e. capillary-to-fiber ratio of ~ 2, *P* < 0.001; and ~ 5 capillaries around the muscle fiber, *P* < 0.001), who possessed higher Mb concentrations (van der Zwaard et al. 2018b). Therefore, the higher capillarization may explain why these cyclists were still able to reach high oxidative capacity within their muscle fibers (van der Zwaard et al. 2016), despite of their lower Mb concentrations. It remains to be determined whether oxygen supply and utilization will further improve when Mb mRNA expression and Mb concentrations are increased in skeletal muscle with already well-developed capillarization.

### Limitations

The present findings suggest that the lower Mb protein concentration in elite cyclists could be partly explained by a lower transcription of Mb within the myonuclei. A lower Mb transcription could potentially relate to the calcineurin/NFAT pathway, PGC-1α and/or IGF-1/AKT/mTOR pathway, but we did not assess these. We encourage future studies to measure all markers involved in these pathways including their interactions, preferably at different time points following acute and chronic exercise. Alternatively, Mb mRNA expression levels could be influenced by microRNAs (miRNAs), small non-coding mRNAs that are involved in post-transcriptional mRNA regulation (Filipowicz et al. 2008; Zacharewicz et al. 2013; Denham et al. 2014; Cui et al. 2016). In particular, muscle-specific miR-499-5p may be involved, which is known to facilitate oxidative muscle fiber gene expression (van Rooij et al. 2009; Wang et al. 2017; Xu et al. 2018; Jiang et al. 2021) by promoting the NFAT/Mef2 pathway (Xu et al. 2018)—via inhibition of Thrap1—and by inhibiting pSox6 (Wang et al. 2017). The miR-499-5p expression has shown to be higher in slow-type *soleus* muscle compared to fast-type *extensor digitorum longus* muscle (Wang et al. 2017) and has been positively related to the Mb expression in porcine muscle (Jiang et al. 2021). Therefore, we speculate that miRNAs (e.g. miR-499-5p) may also play a role in the fiber-type specific findings for Mb in the present study, even though miRNAs were not assessed in this study. Lastly, even though cyclists were measured during off-season and strenuous exercise was avoided before muscle biopsy sampling, we cannot rule out that low Mb protein concentrations could potentially also be due to increased rates of Mb clearance, such as related to muscle damage.

### Practical implications

Myoglobin plays an important role in the O_2_ transport to the mitochondria within the muscle fibers. We observed relatively low Mb concentrations in the muscle fibers of elite cyclists when compared to that of control subjects, which are partly explained by the lower Mb mRNA expression levels. Therefore, athletes and coaches are encouraged to seek training strategies that elevate Mb mRNA levels to enhance their aerobic capacity and endurance performance. The combination of high-intensity exercise in hypoxic conditions seems promising to elevate Mb mRNA expression (Vogt et al. 2001; Brocherie et al. 2018). The optimal training intensity, duration, frequency and hypoxic stimulus remain to be determined in future studies.

## Conclusion

Myoglobin is important in facilitating O_2_ transport to the mitochondria. The average Mb concentration in elite cyclists was surprisingly low compared with physically-active controls. A small proportion of these differences is explained by lower Mb mRNA expression levels and not by a lower myonuclear content. Interestingly, Mb concentration levels were only lower in type I muscle fibers, but not in type II muscle fibers. In summary, regulation of Mb concentration may be partially limited by the lower Mb mRNA expression levels in elite cyclists. It remains to be determined whether cyclists may benefit from training strategies that increase Mb mRNA expression levels using a combination of high-intensity training and hypoxia.

## Data Availability

The dataset supporting the conclusions of this article and the custom-written R scripts that were used to perform the analysis are openly available in the 2022-myoglobin repository at https://github.com/StephanvdZwaard/2022-myoglobin/.

## Abbreviations

ATP: Adenosine triphosphate
Ca^2+^: Calcium
CT: Cycle threshold from the quantitative polymerase chain reaction
DAPI: 4',6-Diamidino-2-phenylindole
FCSA: Fiber cross-sectional area
IGF-1: Insulin-like growth factor 1
LHTL: Live-high-train-low training regimen
Mb: Myoglobin
MDS: Myonuclear domain size
Mef2: Myocyte Enhancer Factor-2
miRNAs: MicroRNAs
mRNA: Messenger RNA
mTOR: Mammalian target of rapamycin
NFAT: Nuclear Factor of Activated T-cell
NRE: NFAT Response Element
O_2_: Oxygen
Pax7: Paired Box 7
PCG-1α: Peroxisome-proliferator-activated receptor-γ coactivator 1
qPCR: Quantitative Polymerase Chain Reaction
RSH: Repeated sprint sessions in hypoxia
RSN: Repeated sprint sessions in normoxia
